# Interactions of Paraoxonase-1 with Pharmacologically Relevant Carbamates

**DOI:** 10.3390/molecules25010211

**Published:** 2020-01-04

**Authors:** Anita Bosak, Aljoša Bavec, Tilen Konte, Goran Šinko, Zrinka Kovarik, Marko Goličnik

**Affiliations:** 1 Institute for Medical Research and Occupational Health, HR 10000 Zagreb, Croatiazkovarik@imi.hr (Z.K.); 2 Institute of Biochemistry, Faculty of Medicine, University of Ljubljana, SI 1000 Ljubljana, Slovenia; aljosa.bavec@mf.uni-lj.si (A.B.); tilen.konte@mf.uni-lj.si (T.K.)

**Keywords:** paraoxonase-1, arylesterase activity, phenyl acetate, *S*-phenyl thioacetate, *p*-nitrophenyl acetate, carbamates, reversible inhibition

## Abstract

Mammalian paraoxonase-1 hydrolyses a very broad spectrum of esters such as certain drugs and xenobiotics. The aim of this study was to determine whether carbamates influence the activity of recombinant PON1 (rePON1). Carbamates were selected having a variety of applications: bambuterol and physostigmine are drugs, carbofuran is used as a pesticide, while Ro 02-0683 is diagnostic reagent. All the selected carbamates reduced the arylesterase activity of rePON1 towards the substrate *S*-phenyl thioacetate (PTA). Inhibition dissociation constants (*K*_i_), evaluated by both discontinuous and continuous inhibition measurements (progress curves), were similar and in the mM range. The rePON1 displayed almost the same values of *K*_i_ constants for Ro 02-0683 and physostigmine while, for carbofuran and bambuterol, the values were approximately ten times lower and two times higher, respectively. The affinity of rePON1 towards the tested carbamates was about 3–40 times lower than that of PTA. Molecular modelling of rePON1-carbamate complexes suggested non-covalent interactions with residues of the rePON1 active site that could lead to competitive inhibition of its arylesterase activity. In conclusion, carbamates can reduce the level of PON1 activity, which should be kept in mind, especially in medical conditions characterized by reduced PON1 levels.

## 1. Introduction

Paraoxonase-1 (PON1) is one of the most common esterases in human serum where it is associated with the high-density lipoprotein (HDL) complex [1]. PON1 prevents oxidation of low-density lipoprotein (LDL) and is associated with risks of developing atherosclerosis, cardiovascular disease, and myocardial infarction [2,3]. The deficiency or inhibition of PON1 activity leads to the development of a wide variety of diseases such as diabetes [4], kidney [5] and liver diseases [6], neurological diseases [7], thyroid disease [8], and cancer [9]. In mammals, PON1 has arylesterase, lactonase, and phosphotriesterase activities [10,11,12], which make it capable of hydrolysing a very wide spectrum of esters, lactones, and certain organophosphorus compounds (OPs), including paraoxon, an oxidized form of the pesticide parathion against which PON has been named [13]. The recently published structure of the G2E6 variant of recombinant PON1 [14] showed that all three hydrolytic activities of the enzymes take place in the same active site, but according to different models and with different conformations of the active site of the enzyme [15,16,17]. Structural and site-directed mutagenesis studies have provided us with much information on the role of individual amino acid residues in the active site of recombinant PON1 [14,15,16], but the exact description of all of the interactions that promote catalysis with this enzyme still remains elusive. However, its lactonase and arylesterase activities are proposed to be hydroxide-ion generated via general base catalysis by H115–H134 dyad [14,15], and quantitative structure-activity relationships [11] and recent exploration of PON1 catalysis through solvent kinetic isotope effects and phosphonate-based isosteric analogue of tetrahedral reaction intermediate [18] have suggested that this mechanism of the PON1-catalysed reaction pathway might be correct.

Although many insecticides are esters of carbamic acid, and technical monographs haves suggested that the hydrolysis of ester bonds of carbamates plays an important role in the detoxification of these compounds, detailed studies about enzymes hydrolysing carbamates in vertebrates are not available. Certain carbamate hydrolysing activities are associated to serum albumin and by carboxylesterase depending on the structure of carbamate and on the species origin of the carboxylesterase [19]. In the literature, there are very few data related to the role of PON1 in the metabolism of carbamates. It was demonstrated that carbamate pesticides oxamyl and aldicarb are partially metabolised by PON1 in human liver [20]. Since PON1 is involved in the metabolic or detoxification pathway of certain OP compounds [12,21], and, due to its ability to hydrolyse esters, PON1 could also be expected to exhibit a certain affinity to carbamates. Some studies have described the decrease of PON1 activity in the presence of certain carbamates [20], while in others, no effect of carbamates on PON1 activity was found [22,23,24,25]. The main mode of action of OPs and carbamates in vertebrates is the inhibition of acetylcholinesterase (AChE) and the related enzyme butyrylcholinesterase (BChE) which lead to overstimulation of the cholinergic nervous system [26,27]. Research aimed at determining possible effects of carbamates on PON1 activity would contribute to the characterization of the role of individual residues in the mechanism of hydrolysis of various substrates by PON1, and to its possible role in the biotransformation of carbamic acid derivatives. More recently, the carbamate group has become an important part of the structure of many FDA-approved drugs, having antitumor, antibacterial, antifungal, antimalarial, antiviral, anti-inflammatory, anticonvulsant, or anti-helminthic effects on a wide range of targets [28,29]. Additionally, the need for research aimed at clarifying the metabolic path of carbamates with a diverse structure is underlined by the development of carbamate-based prodrugs [30] whose activation would depend on the esterases of the human serum and by the fact that PON1 is considered as a scavenger enzyme in nervous system poisoning by OP compounds.

**Figure 1:**
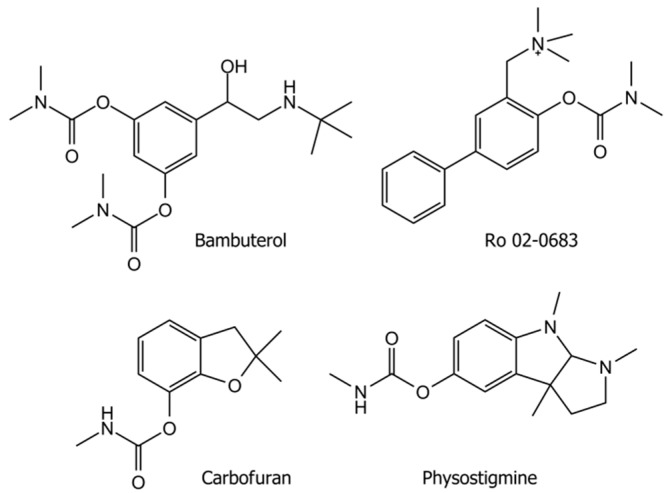
Structures of selected carbamates.

The aim of the present study was thus to determine whether selected carbamates inhibit the activity of the G2E6 variant of recombinant PON1. We have therefore determined the affinity of PON1 towards four carbamates used in different applications (Figure 1), i.e., bambuterol, which acts as a bronchodilator, physiostigmine as a drug for treating glaucoma and delayed gastric emptying, carbofuran, that is in use as a pesticide, and Ro 02-0683, used as a diagnostic reagent for phenotyping human serum butyrylcholinesterase. We have also rationalized the *in vitro* affinity for enzyme-inhibitor complex formation by molecular modelling.

## 2. Results

### 2.1. rePON1 Arylesterase Activity

Hydrolysis of phenyl acetate (PA), *p*-nitrophenyl acetate (PNPA) and of *S*-phenyl thioacetate (PTA) was measured spectrophotometricaly at 270, 405, and 412 nm, respectively. The initial velocity of product formation was expressed in ΔA/min (change of absorbance per minute) and determined at six substrate concentrations over the range 100 µM to 5 mM for PA and PTA, and to 3 mM for PNPA.

**Figure 2:**
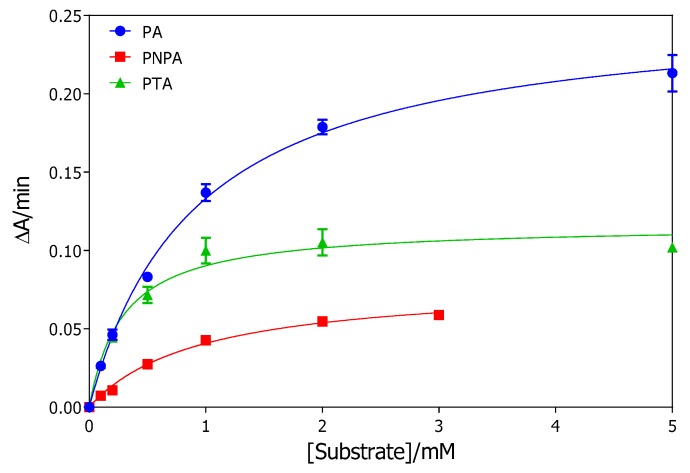
The rates of hydrolysis of phenyl acetate (PA), *p*-nitrophenyl acetate (PNPA) and of *S*-phenyl thioacetate (PTA) by rePON1. Points represent average values of three independent experiments corrected for the spontaneous hydrolysis of the corresponding substrate. Solid lines are curves calculated by the Michaelis–Menten equation and the parameters given in Table 1.

The rePON1-catalyzed rates of substrate hydrolysis as a function of concentration follow the Michaelis–Menten kinetics, as illustrated in Figure 2, and the corresponding catalytic constants K_m_ and V_max_ were calculated from the theoretical curves that fit the Michaelis–Menten model equation (Table 1). The extinction coefficients of the phenol, TNB¯ anion, and *p*-nitrophenolate anion used for the calculation were 1,310 [31], 14,150 [32] and 12,750 M^−1^∙cm^−1^ [33], respectively. Since the enzyme dilution ratios were not equal for all substrates, the limiting specific activity (SA_max_) of rePON1 per mL of its stock solution under substrate saturation conditions was also determined for comparison (Table 1).

The Michaelis constants showed that the G2E6 variant of rePON1 has the same affinity (1/*K*_m_) for PA as for PNPA, while its affinity towards PTA is three times higher. The specific activities show that rePON1 hydrolyses PA about five times faster than does PTA and about 30 times faster than PNPA.

The pseudo-first order rate constants for spontaneous hydrolysis of substrates were also evaluated according to Equation (1), and the results are given in Table 2.

The highest pseudo-first order rate constant was that for the hydrolysis of PNPA, while the hydrolysis of PA was almost negligible and occurred significantly only at concentrations higher than 2 mM.

### 2.2. The Effect of Carbamates on rePON1 Activity

#### 2.2.1. Dissociation Constants Determined from the Initial Rates

**Figure 3:**
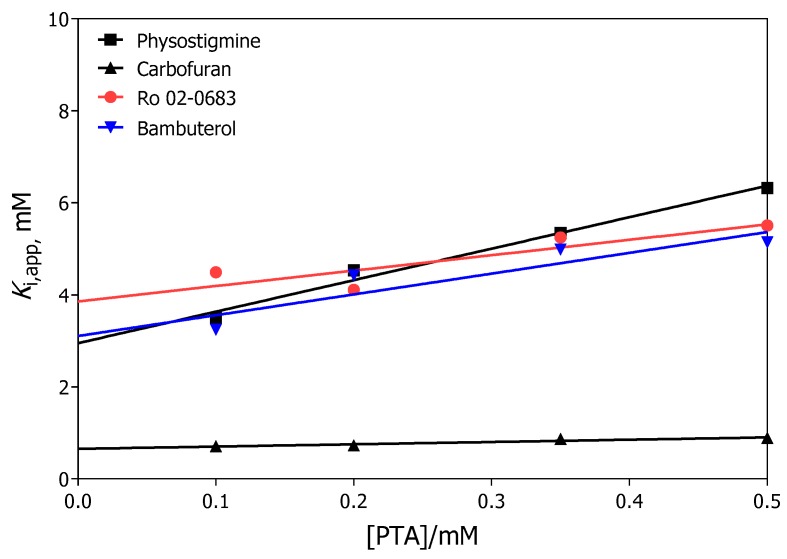
Inhibition of rePON1 hydrolysis of PTA by selected carbamates evaluated by linear regression from at least three experiments.

The effect of carbamate esters on rePON1 arylesterase activity was determined by measuring its activity in the absence and the presence of carbamates. As a measure of the inhibition potency of the tested carbamates, the dissociation constants (± standard error of mean) of the enzyme-inhibitor complex (*K*_i_) were determined. Four substrate concentrations in the range of 100 to 500 µM were used; the highest being about 70% of the maximal enzyme activity. For each substrate concentration, three concentrations of carbamate that inhibited PON1 in the range of 20%–50% were chosen. Higher concentrations of carbamates could not be used due to the low solubility of carbamates. Linear regression analysis using the Hunter–Downs equation, i.e., Equation (2), yields the kinetic constants *K*_i_ and *K*_s_ (Table 3). The y-intercepts in Figure 3 indicate the enzyme-inhibitor dissociation constants (*K*_i_) for various carbamates, while the slopes determine the ratio *K*_i_/*K*_s_, and, consequently, the enzyme-substrate dissociation constant *K*_s_.

The linear dependence of *K*_i,app_ on the substrate concentration (Figure 3) for all the carbamates tested, and the values of the determined *K*_i_ constants (Table 3) for enzyme-carbamate dissociation close to the *K*_s_ value of PTA, show that the inhibitors compete with PTA for binding at the catalytic site of the enzyme.

#### 2.2.2. Stability of the G2E6 Variant of rePON1

**Figure 4:**
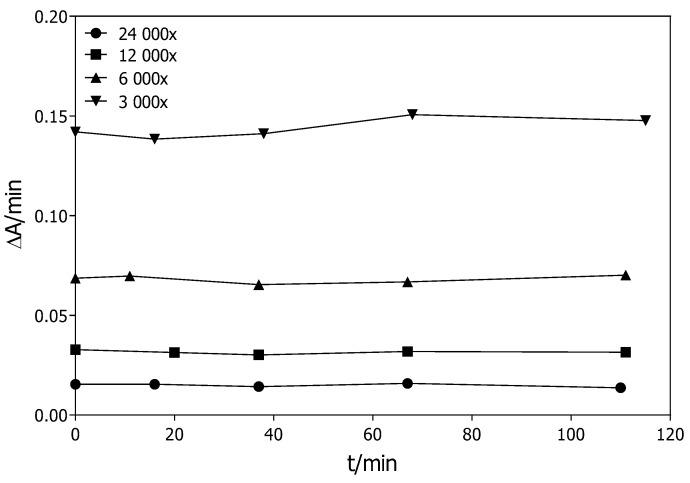
Stability of the activity of the G2E6 variant of rePON1 diluted 3000, 6000, 1200 and 24,000 times.

The stability of the activity of the G2E6 variant of rePON1 diluted 3000, 6000, 12,000, and 24,000 times in Tris/HCl buffer supplemented with 1.0 mM CaCl_2_, pH = 8.0 with 1.0 mM PA as substrate at 25 °C is shown in Figure 4.

#### 2.2.3. Dissociation Constants Determined from the Progress Curves

**Figure 5:**
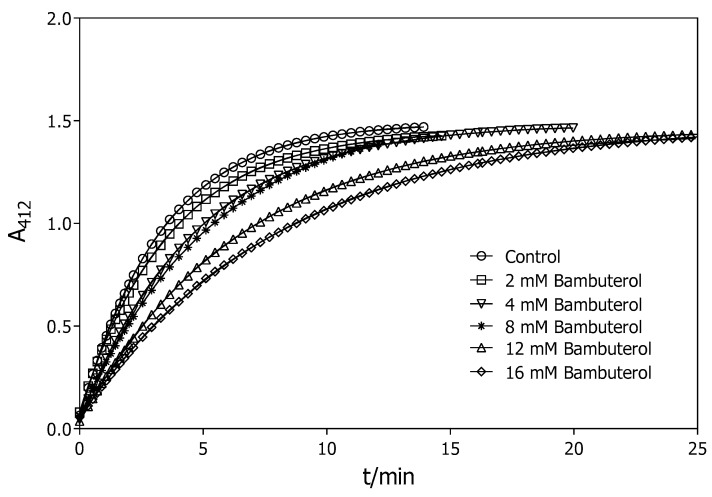
Progress curves for the hydrolysis of PTA by rePON1 in the absence (control) and in the presence of bambuterol (2 mM, 4 mM, 8 mM, 12 mM and 16 mM). The full lines represent theoretical curves calculated using Equation (3).

Progress curves for the hydrolysis of PTA by rePON1 in the absence and presence of all tested carbamates were measured. As an example, the progress curves with bambuterol are shown in Figure 5.

**Figure 6:**
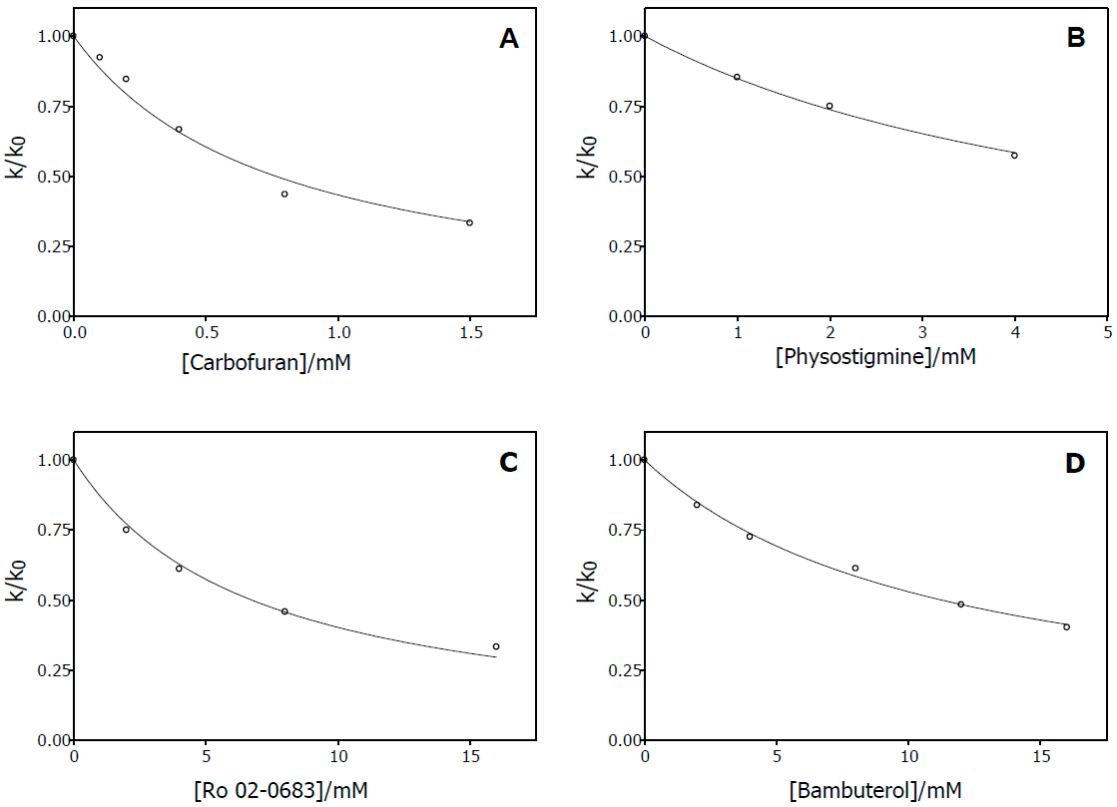
Dependence on inhibitor concentration of normalized *k*/*k*_0_ values for carbofuran (**A**), physostigmine (**B**), Ro 02-0683 (**C**) and bambuterol (**D**). Lines represent theoretical curves calculated using Equation (5).

The curve for PTA hydrolysis in the absence of carbamates reached a plateau that corresponds to 50 µM PTA. This plateau was reached on raising the concentrations of the carbamates. Rate constants were calculated from progress curves in the presence (*k*) and in the absence of carbamates (*k*_0_). As a result, the dissociation constants of the enzyme-inhibitor complex (*K*_i_) (Table 4) were evaluated from the ratio, *k*/*k*_0,_ of rate constants versus inhibitor-concentration (Figure 6) as described by Equation (5).

### 2.3. Molecular Modelling of the PON1-carbamate Complex

Molecular docking was conducted using the structure of PON1 (PDB ID 1V04) [14] to reveal the key interactions of carbamates that appear to be important for the inhibition of rePON1 arylesterase activity. Phosphate ions and water molecules were removed from the crystal structure before modelling. All the interactions in the PON1 active site are listed in Table 5.

**Figure 7:**
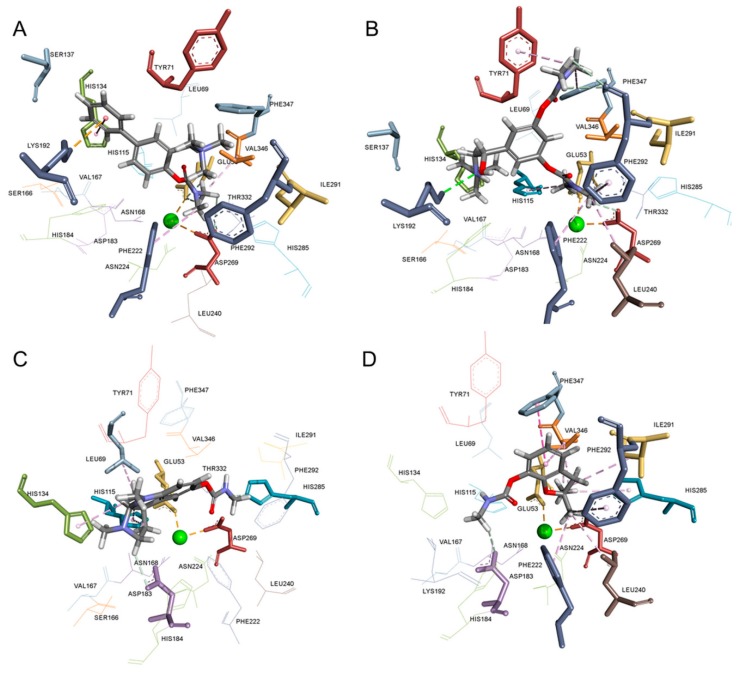
Simulation of the dissociation complex of PON1 and Ro 02-0683 (**A**), bambuterol (**B**), physostigmine (**C**), and carbofuran (**D**). Interactions are presented with green (H-bonds), orange (cation-π interaction), and purple (hydrophobic interactions) dashed lines. Residues Tyr71, His134, Asn168, Phe222 and Val346 are singled out as important for the stabilisation of substrate PTA.

In general, all modelled PON1-carbamate complexes were positioned close to the catalytic Ca^2+^, forming hydrogen bonds and/or π–π interactions with the residues of the catalytic dyad (His 115 and His134) or with residues Glu53 and Asp269 that are involved in the ligation of catalytic Ca^2+^ (Figure 7).

A comparison of the size of the carbamates and their positioning in the active site revealed that the more voluminous carbamates, bambuterol (360 Å^3^) and Ro 02-0683 (318 Å^3^), form hydrogen bonds with Asp269 that are involved in the ligation of catalytic Ca^2+^. This may explain the competitive inhibitory effects of bambuterol and Ro 02-0683 on rePON1 activity. For the smaller carbamates, physostigmine (267 Å^3^) and carbofuran (211 Å^3^), the hydrogen bond formed with Asp183 appears to be crucial for the observed competitive inhibitory effect. Further, the rigid structure of carbofuran may contribute to the higher PON1 affinity. In other words, the more flexible ligand, bambuterol, needs to adopt the binding conformation that would be complementary to the conformation of the mobile loop positioned above the catalytic site.

## 3. Discussion

PON1 hydrolyses a wide range of substrates, such as esters, thioesters, phosphotriesters, lactones and thiolactones, as well as 5-thioalkyl butyrolactones [34]. The main physiological role of PON1 appears to be the hydrolysis of oxidized lipids in macrophages that yields lyso-glycerophosphatidyl lipids in cell membranes [35] and provides protection against protein *N*-homocysteinylation [36]. The highest activities observed in vitro are against the synthetic substrates PA for esterase and dihydrocumarine for lactonase activity [10,11] that have no physiological relevance. As PON1 shows several promiscuous reaction activities, it might be expected that it could also catalyse the hydrolysis of carbamate bonds. Carbamates are semi-substrates for cholinesterases, although they act as inhibitors because of their relatively high affinity binding in the reaction site, resulting in active serine carbamylation followed by slow reactivation with a low decarbamylation rate [37]. The ability of rePON1 to hydrolyse carbamates was studied in this work by using the competing kinetics method based on analysis of the kinetics of competition of pairs of two substrates in such a way that the hydrolysis of only one substrate was measured at a time [38,39]. Although a carbamate, as an ‘invisible’ semi-substrate, could be hydrolysed, it behaves as a competing ligand towards ‘visible’ substrates such as PA, and its effect on the enzyme activity is thus similar to the effect produced by a reversible competitive inhibitor. In that case the ratio of the specificity rate constants (*V*_max_/*K*_m_) for ‘invisible’ and ‘visible’ substrates, i.e., competition matrix R, is significantly lower than 1 (R << 1) [38,39].

PA is the most commonly used substrate for measuring the arylesterase activity of PON1. However, it is based on measuring the increase of phenol concentration at λ = 270 nm, the part of the spectrum in which compounds with an aromatic ring also exhibit maximum absorption. This became a problem in the case of the carbamates used in this study (Figure 1), since the increased phenol concentration during the hydrolytic process interferes with the absorbance of their aromatic rings. For this reason, the other two aryl esters, PNPA and PTA, that are also PON1 substrates for which PON1 activity could be measured spectrophotometrically in the visible part of the spectrum, were tested as possible replacements for PA [11,40,41,42,43]. Evaluated kinetic parameters for those substrates are given in Table 1 and Table 2. The ratios of rePON1 catalytic proficiencies (SA_max_/*K*_m_)/*k*_sp_ for PA: PTA: PNPA are approximately 660: 80: 1, despite the fact that 4-nitro phenoxide (p*K*_a_ (4-nitro phenol) = 7.2) and thiophenoxide (*pK_a_* (thiophenol) = 6.6) anions are better leaving groups during the hydrolysis than the phenoxide (*pK_a_* (phenol) = 10) anion. We thus estimate that the hydrolysis of PA is an energetically favoured process, by five-fold (=R∙T∙ln(660/80)) and by 16-fold (=R∙T∙ln(660/1)) kJ/mol relative to PTA and PNPA, respectively. This finding is in accordance with previous published results [15,18,38] and with the reaction mechanism model that proposes that the catalytic power of rePON1 can be mostly rationalised by concerted two-proton exchange referred to the histidine shuttle dyad. Considering a 20-fold higher catalytic efficiency (SA_max_/K_m_) and an 80-fold higher catalytic efficiency (SA_max_/*K*_m_)/*k*_sp_ of rePON1 towards PTA compared to that for PNPA, we have selected PTA as a replacement for PA in the following inhibition study on PON1 and carbamates.

Inhibition constants, *K*_i_, were first estimated from the initial rates of non-inhibited and inhibited measurements for four substrate concentrations in the range of 100–500 µM. The highest substrate concentration was about 70% of the maximum enzyme activity. For each substrate concentration, three concentrations of carbamate that inhibited PON1 in the range of 20%–50% were chosen. Higher concentrations of carbamate needed to achieve about 80% inhibition of PON1 activity could not be used because of the limited solubility of carbamates in methanol or in water. Additionally, the use of higher carbamate concentrations would increase the methanol concentration in the enzyme activity assay. The enzyme-inhibitor complex dissociation constants were determined from the Hunter–Downs plot (Figure 3, Table 3). rePON1 displayed similar affinities towards Ro 02-0683, bambuterol and physostigmine, while that toward carbofuran was about four times higher. However, the affinities of rePON1 towards the tested carbamates were 2 to 13 times lower than that toward the *K*_m_ value of 0.29 mM for PTA. The appropriate affinities, *K*_s_, of rePON1 toward PTA varied significantly in the presence of different carbamates.

We therefore decided to evaluate the inhibition constants from the progress curves because this methodology has been to be a powerful tool for the determination of relevant kinetic constants for other esterases [44]. Long-time experiments showed that diluted rePON1 would be stable for up to 120 min under our experimental conditions (Figure 4). These results are in accordance with those in a previous study, wherein it was shown that an EDTA sensitive esterase from human sera diluted 2160 times in Tris/HCl containing 1.0 mM CaCl_2_, pH = 8.4 was stable at room temperature for up to 30 min [45].

**Scheme 1:**
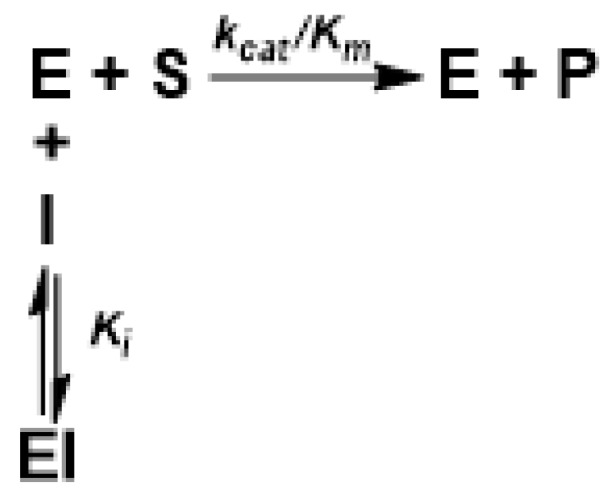
The model of competitive inhibition at substrate unsaturated conditions.

The progress curves for the hydrolysis of PTA with a starting concentration of 0.05 mM significantly below the *K*_m_ (0.29 mM) showed that, with increased concentrations of the carbamates, the plateau of the reaction was reached later, as illustrated in Figure 5. Such a pattern of enzyme activity changes in the presence of carbamates indicates that they bind to PON1 by forming noncovalent interactions within the active site, thereby competing with binding of the substrate, PTA. This result is consistent with those in a previous study where an approximately 10% decrease in rabbit serum activity toward PA was observed and remained at the same level after 30 min incubation with physostigmine [46]. In other words, since the specificity rate constants (*V*_max_/*K*_m_) for carbamates are well below the value of the same parameter for PTA, carbamates act either as reversible inhibitors or as extremely poor ‘invisible’ competing substrates [38,39]. We propose a simple reaction scheme (Scheme 1) that assumes binding of carbamates to the active site PON1 that enables simultaneous fitting of both non-inhibited and inhibited experimental curves. The subsequent fitting curves followed closely the experimental points, indicating that the proposed mechanism describes well the mode of binding of carbamate to the active site of PON1. Although all the carbamates have *K*_i_ constants in the millimolar range (see Table 4), and although those obtained from progress curves for all carbamates except carbofuran are higher than those evaluated from initial rate calculations (Table 3), the time-course kinetic measurements provide additional support for our results obtained from initial rate experiments; i.e., that carbamates are poor rePON1 inhibitors. In addition, the best known reversible PON1 inhibitor, 2-hydroxyquinoline, has *K*_i_ constants for the most familiar substrates in micromolar range [11]. It seems that the size and rigidity of the compounds is crucial for adopting the optimal position into the PON1 active site for achieving interactions with amino acid residues that stabilize the compound-PON1 complex [16].

**Figure 8:**
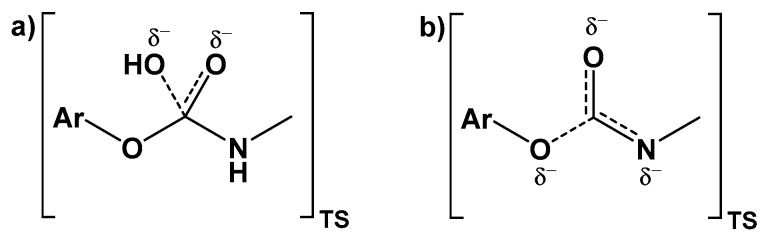
Proposed transition states on two possible reaction pathways for hydrolysis of an aryl carbamate ester: (**a**) via an energetically less favoured addition-elimination (B_Ac_2) mechanism, or (**b**) via the energetically more favoured elimination-addition (E1cB) mechanism. The latter mechanism is not possible for carbamate esters lacking an N-H group.

To the best of our knowledge, it is shown here for the first time that carbamate affects PON1 activity. Although we have proposed a reversible competitive mode of their action, it cannot be disproved that weak hydrolysis of carbamate esters also proceed in the rePON1 active site, since the reversible competitive inhibition behaviour of carbamates can be the only apparent effect of the very low competition matrix R [38]. However, it appears that the aryl carbamate ester hydrolysis reaction probably occurs predominantly via an elimination-addition (E1cB) mechanism for N-H carbamates (physostigmine and carbofuran, Figure 1), while the disfavoured addition-elimination (B_Ac_2) pathway, involving a tetrahedral intermediate could only operate effectively for carbamate esters lacking an N-H group (bambuterol and Ro 02-0683, Figure 1) [47]. It is estimated that, for hydrolysis of the aryl carbamates, there is a free energy difference between spontaneous E1cB and B_Ac_2 transition states (Figure 8) of more than 50 kJ/mol [48].

As there are no significant differences in the inhibition constants *K*_i_ for the two types of carbamates, we may conclude that the rate-limiting step in the enzyme-catalysed reaction in the PON1 active site is coordinated with the acid-base activation of a water molecule that attacks the carbonyl carbon atom, supporting the disfavoured addition-elimination (B_Ac_2) pathway that involves a tetrahedral intermediate. Moreover, the binding energy of up to 50 kJ/mol may not be available in the rePON1 active site, since all the carbamates tested bind with low affinities. It thus appears that this enzyme is not able to change the reaction pathway to one through another transition state. However, additional studies will have to be carried out to further identify the mechanism of the rePON1-catalysed reaction pathway for the hydrolysis of carbamate esters.

## 4. Materials and Methods

### 4.1. Chemicals

The substrates *S*-phenyl thioacetate (PTA), phenyl acetate (PA) and *p*-nitrophenyl acetate (PNPA) were purchased from Alfa Aesar (Ward Hill, MO, USA), Sigma Aldridge (St. Louis, MO, USA) and Polyscience (Warrington, PA, USA), respectively. The carbamates Ro 02-0683 and bambuterol were purchased from PolyScience Inc. (Warrington, PA, USA) and Astra Draco (Lund, Sweden), while physiostigmine, carbofuran, and 5,5′-dithiobis-(2-nitrobenzoic acid) (DTNB) were purchased from Sigma Aldridge (St. Louis, MO, USA). Nickel-nitrylotriacetic acid (Ni-NTA) was from Qiagen (Hilden, Germany); all other chemicals for the expression and purification of rePON1 were from Sigma Aldridge (St. Louis, MO, USA). All chemicals for the preparation of Tris/HCl buffer and solvents were purchased from commercial sources. Carbamates Ro 02-0683 and bambuterol were dissolved in water, physostigmine and carbofuran in methanol.

### 4.2. Recombinant PON1 Expression and Purification

The G2E6 variant of recombinant PON1 (rePON1) was used as a source of enzyme. RePON1 protein was expressed and purified in the Escherichia coli bacterial system according to the procedures reported previously [49] with minor modifications [18]. A single colony obtained after transformation with plasmid pET32b(+)-rePON1 into Origami B(DE3)pLysS cells was used to inoculate 10 mL of LB medium with 100 µg/mL ampicillin, 25 µg/mL chloramphenicol and 1 mM CaCl_2_ and the culture was grown at 37 °C for 17 h. Then, 500 mL of LB medium containing 100 µg/mL ampicillin, 25 µg/mL chloramphenicol and 1 mM CaCl_2_ was inoculated with 5 mL of overnight culture and grown at 37 °C to an OD600 of 0.7. Expression of the rePON1 variant was induced by adding 1 mM isopropyl β-d-1-thiogalactopyranoside (IPTG), and the culture was grown for 17 h at 25 °C. The cells were harvested by centrifugation at 6000× *g* for 15 min and the pellet stored overnight at −20 °C. The cells were resuspended in 30 mL of lysis buffer (50 mM Tris, pH = 8.0, 1 mM CaCl_2_ and 0.1 mM dithiothreitol (DTT) supplemented with 1 µM pepstatin A, 1 mM phenylmethylsulfonyl fluoride (PMSF) and 0.03% *n*-dodecyl-β-d-maltopyranoside (C_12_-maltoside), then lysed by sonification. The lysate was centrifuged at 10,000× *g* for 10 min and the supernatant stirred for 1 h at 4 °C. After centrifugation at 20,000× *g* for 20 min, the soluble fraction was treated with ammonium sulphate (55%, *w*/*v*, at 0 °C). The precipitate was centrifuged at 10,000× *g* for 15 min, resuspended and dialyzed twice against lysis buffer supplemented with 0.01% C_12_-maltoside. After dialysis, the protein was added to Ni-NTA resin, and the mixture shaken gently overnight at 4 °C. The resin was first washed with lysis buffer with 0.03% C_12_-maltoside, then with 10 and 20 mM imidazole in lysis buffer with 0.03% C_12_-maltoside. It was finally eluted with 150 mM imidazole in lysis buffer with 0.03% C_12_-maltoside. Fractions with the highest rePON activity were pooled, dialyzed and purified further by ion-exchange chromatography. The protein was applied on a 5 mL HighTrap Q HP column (GE Healthcare, City, Marlborough, MA, USA) with a linear gradient from 26% to 33% of buffer B (20 mM Tris, pH = 8.0, 1 mM CaCl_2_, 0.1 mM DDT, 0.03% C_12_-maltoside, 1 M NaCl) in buffer A (buffer B without 1 M NaCl). Fractions with the highest rePON activity were analysed on an 11% SDS–PAGE gel, pooled, dialyzed against buffer A and concentrated. Finally, sodium azide (0.02%) was added and the protein stored at −70 °C.

The purity of the rePON1 (95%) was finally assessed by SDS-PAGE, and its concentration determined using the Bradford assay (Bio-Rad, Hercules, CA, USA). A stock solution of 1.9 mg/mL rePON1 was used for all measurements, except for progress curve measurements where the stock solution of 0.2 mg/mL rePON1 was used.

### 4.3. Determination of the Catalytic Constants of rePON1 from Initial Rate Measurements

Hydrolysis of PA, PNPA, and PTA was measured in 50 mM Tris/HCl buffer (pH = 8.0) containing 1 mM CaCl_2_ at 25 °C using a Cary 300 spectrophotometer (Varian, Australia). For PA the increase of phenol was measured at 270 nm, for PNPA *p*-nitrophenol was measured at 405 nm and for PTA, the release of thiophenol was measured in the presence of DTNB, as a thiol reagent, at 412 nm, using the Ellman method [50]. Stocks of PA, PTA and PNPA were prepared in MeOH; 6.0 mM DTNB was dissolved in 10 mM phosphate buffer, pH = 7.4. The concentration of phosphate in the final reaction mixture containing DTNB was 0.5 mM. The final dilutions of rePON1 stock solution were 3000 times for PA and PNPA activity measurements and 15,000 times for PTA. The percentage of methanol was kept at 1% in all reaction mixtures.

The rates (*v*) of spontaneous hydrolysis for all three substrates were also measured, and the pseudo-first order rate constants (*k*_sp_ = *k*_H2O_∙[H_2_O]) were calculated for each substrate as:

(1) $$v=\frac{\Delta \left[P\right]}{\Delta t}=k_{sp}\cdot \left[S\right]$$

where Δ[*P*] is the increase in phenol, *p*-nitrophenolate and TNB¯ anion concentrations over time Δt, and [S] is the initial PA, PNPA or PTA concentration. Consequently, rePON1 activity was corrected for spontaneous hydrolysis of the substrate.

The catalytic constants *K*_m_ (the Michaelis constant) and *V*_max_ (the maximal rate) were determined by applying the Michaelis–Menten equation [51] using the GraphPad Prism 6 software (GraphPad Software, San Diego, CA, USA).

### 4.4. Determination of the rePON1-carbamate Complex Dissociation Constants

RePON1 activity was determined from the measurement of initial velocities in the absence (*v*_0_) and in the presence (*v*_i_) of various carbamate concentrations [I], using PTA as a substrate at a given concentration [S]. For each substrate concentration, the apparent dissociation constant (*K*_i,app_) was calculated by linear regression analysis using the Hunter–Downs equation [52] as:

(2) $$K_{i,app}=\frac{v_{i}\cdot \left[I\right]}{\left(v_{0}-v_{i}\right)}=K_{i}+\frac{K_{i}}{K_{S}}\cdot \left[S\right]$$

where *K*_i_ stands for the enzyme-carbamate dissociation constant and *K*_S_ for the enzyme-substrate dissociation constant. Kinetic constants were determined using GraphPad Prism 6 software (GraphPad Software, San Diego, CA, USA).

### 4.5. Stability of the G2E6 Variant of rePON1

Stability was studied in Tris/HCl buffer supplemented with 1.0 mM CaCl_2_ at pH = 8.0. The enzyme activity was measured with 1.0 mM PA as substrate at 25 °C. The enzyme stock solution was diluted 30, 60, 120 and 240 times and kept on ice for up to 120 min before the activity measurements. At given time intervals, aliquots (10 µL) were withdrawn, added to buffer containing substrate, and time-dependent increase in absorbances were measured in cuvettes with a final reaction mixture volume of 1 mL. Final dilutions of rePON1 were 3000, 6000, 12,000 and 24,000 times.

### 4.6. Determination of the Inhibition Constants of Carbamates from Progress Curve Measurements

rePON1 activity was determined from progress curves for the measurement of hydrolysis of 50 µM PTA (about 6 times below the value of *K*_m_) in the absence and in the presence of various concentrations (range 0.2–8.0 mM, depending on the compound) of carbamates using the modified Ellman’s method [50]. The measurements were performed on a Genesys 10S spectrophotometer (Thermo Fisher Scientific, Pittsburgh, PA, USA). The hydrolysis of PTA was followed from zero to the plateau for up to 30 min (i.e., until the change in absorbance became zero). The concentration of DTNB at the beginning of each measurement was 1.0 mM and measurements were performed at room temperature. The 2.0 mM stock solution of DTNB was prepared in Tris/HCl, 50 mM, pH = 8.0, supplemented with 1.0 mM CaCl_2_. The progress curves were analysed using GraphPad Prism 6 software (GraphPad Software, San Diego, CA, USA).

For the analysis of progress curves, the model of competitive inhibition at substrate unsaturated conditions was used, as shown in Scheme 1.

In this scheme, E and EI represent the free enzyme and enzyme-inhibitor complex at substrate concentrations significantly below *K*_m_, while S and P stand for substrate and product. Simultaneous fitting of the hydrolysis of PTA by rePON1 in the absence and presence of carbamates allowed evaluation of the first-order rate constant (*k*) using a single exponential Equation (3):

(3) $$A_{412}=\Delta A_{412}\cdot \left(1-e^{-k\cdot t}\right)+A_{0}$$

where A_412_ represents the measured time-dependent absorbance, A_0_ is the initial (baseline) absorbance at time zero, and ΔA_412_ stands for the difference between the fitted plateau and baseline values. The first-order rate constant *k* is an inhibitor concentration dependent quantity according to Equation (4):

(4) $$k=\frac{V_{max}}{K_{m}\cdot \left(1+\left[I\right]/K_{i}\right)}$$

Normalization of the first-order rate constant *k* allows evaluation of the inhibition constant *K*_i_ using Equation (5):

(5) $$\frac{k}{k_{0}}=\frac{1}{\left(1+\left[I\right]/K_{i}\right)}=\frac{K_{i}}{\left(K_{i}+\left[I\right]\right)}$$

where the rate constant *k*_0_ is calculated from the progress curve in the absence of carbamates.

### 4.7. Molecular Modelling of the rePON1-carbamate Complex

The three-dimensional structure of rePON1 PDB code 1V04 [14] was used for molecular modelling. Carbamate structures were modelled and minimized using the MMFF94 force field implemented in ChemBio3D Ultra 12.0 (PerkinElmer, Inc., Waltham, MA, USA). Discovery Studio 2017 R2, with the CDOCKER docking protocol, using a CHARMM force field (BioVia, San Diego, CA, USA), generated 20 docking poses for each carbamate in the active site gorge of rePON1, as described earlier [53]. Poses were scored and ranked according to the calculated CDOCKER energy for interactions between carbamate and rePON1 active site residues (i.e., hydrogen bonds, π–π interactions, cation–π interactions and electrostatic interactions).

## 5. Conclusions

In this study, it has been shown that selected carbamates can reduce PON1 arylesterase ability to hydrolyse PTA as a substrate. This reduction is a result of the competition of carbamates and PTA for binding to the PON1 active site, forming non-covalent interactions with relevant residues. Although the carbamates tested were not potent PON1 inhibitors, the possibility of inhibition by carbamates should be kept in mind, especially under conditions characterized by reduced PON1 activity levels.

## Figures and Tables

**Table 1:** Catalytic constants *K*_m_ and *V*_max_. The rates of rePON1 hydrolysis of phenyl acetate (PA), *p*-nitrophenyl acetate (PNPA) and *S*-phenyl thioacetate (PTA) were determined from at least three experiments, and in each case all of the activities were measured in duplicates.

Substrate	*K*_m_ (mM)	ΔA_max_/min	*V*_max_ (µM∙min^−1^) *	SA_max_ (µmol∙min^−1^∙mL^−1^)
PA	0.92 ± 0.06	0.255 ± 0.006	194 ± 5	582 ± 15
PNPA	0.95 ± 0.10	0.079 ± 0.003	6.2 ± 0.2	18.6 ± 0.6
PTA	0.29 ± 0.04	0.116 ± 0.004	8.2 ± 0.3	123 ± 5

*For recalculating rates from ΔA/min data the following relationship was used: $\frac{\Delta A}{\min}=\varepsilon \cdot l\cdot \frac{\Delta c}{\min}=\varepsilon \cdot l\cdot v$, where ε, l, and v stand for the extinction coefficient, cuvette length and rate of product formation in the reaction mixture, respectively.

**Table 2:** The pseudo first-order rate constants of spontaneous hydrolysis of PA, PNPA and PTA determined in 50 mM Tris/HCl buffer, pH = 8.0, containing 1.0 mM CaCl_2_ at 25 °C.

	PA	PNPA	PTA
*k*_sp_ (10^−3^ min^−1^)	0.61 ± 0.07	12.4 ± 0.7	3.5 ± 0.3

**Table 3:** Reversible inhibition of rePON1 by the carbamates tested in the presence of PTA at 25 °C. Kinetic parameters *K*_i_ and *K*_s_ (± standard error of mean) were evaluated with Hunter–Downs Equation (2) from at least three experiments.

Carbamate	*K*_i_ (mM)	*K*_s_ (mM)
Ro 02-0683	3.9 ± 0.6	1.2 ± 0.7
Bambuterol	3.1 ± 0.4	0.6 ± 0.2
Physostigmine	3.0 ± 0.6	0.4 ± 0.1
Carbofuran	0.65 ± 0.05	1.3 ± 0.4

**Table 4:** Reversible inhibition of rePON1 hydrolysis of PTA by selected carbamates (*K*_i_ ± standard error of mean) evaluated using the progress curves from at least three experiments.

Carbamate	*K*_i_ (mM)
Ro 02-0683	11.3 ± 0.4
Bambuterol	6.8 ± 0.4
Physostigmine	5.6 ± 0.2
Carbofuran	0.77 ± 0.07

**Table 5:** PON1-carbamate complex binding interactions with the active site gorge residues evaluated by molecular docking. The crystal structure of PON1 used was PDB ID 1V04.

Carbamate	Type of Interaction		
	H-bond	π Interactions *	Aliphatic Non-polar Interactions
Ro 02-0683	Glu53, Asp269	His134, Lys192, Phe222	Val346
Bambuterol	Lys192, Asp269, Phe292	Tyr71, His115, Phe222, Phe292, Phe347	Leu240
Physostigmine	Asp183, His285	His115, His134	Leu69
Carbofuran	Asp183	Phe222, His285, Phe292, Phe347	Leu240, Ile291, Val346

* π—Interactions refers to π–π, CH-π and cation-π interactions.
